# Supplementary material for: Connectivity assessment and prioritization of urban grasslands as a helpful tool for effective management of urban ecosystem services
Source: PLoS One. 2020 Dec 28;15(12):e0244452. doi: 10.1371/journal.pone.0244452 (PMC7769447; doi:10.1371/journal.pone.0244452)
Supplement: S1 Table — Values above the diagonal of the matrix are shown. Only significant correlations are presented. (PDF) [file pone.0244452.s001.pdf]

S1 Table. Spearman rank correlations between patch area (area), dIIC (dIIC) values, and dIIC components (intra, flux, connector) for distance thresholds (2, 20, 44, 100, and 1000 m). Values above the diagonal of the matrix are shown. Only significant correlations are presented.

	2_dIIC	2_intra	2_flux	2_connector	20_dIIC	20_intra	20_flux	20_connector	44_dIIC	44_intra	44_flux	44_connector	100_dIIC	100_intra	100_flux	100_connector	1000_dIIC	1000_intra	1000_flux	1000_connector
area	0.995	0.998	0.088	-0.060	0.758	0.998	0.327	0.511	0.558	0.998	0.464	0.494	0.795	0.998	0.818	0.488	0.989	0.997	0.989	0.486
2_dIIC		0.995	0.139	-0.043	0.763	0.995	0.334	0.515	0.561	0.995	0.467	0.496	0.792	0.993	0.815	0.485	0.985	0.992	0.985	0.484
2_intra			0.088	-0.060	0.758	1.000	0.327	0.511	0.559	1.000	0.465	0.494	0.795	0.998	0.818	0.488	0.989	0.997	0.989	0.486
2_flux				0.365	0.140	0.088	0.162	0.148	0.124	0.088	0.120	0.134	0.102	0.089	0.102	0.104	0.090	0.089	0.090	0.111
2_connector						-0.060	0.045	0.045		-0.059		0.056	-0.042	-0.059	-0.042		-0.060	-0.055	-0.060	
20_dIIC						0.758	0.757	0.800	0.677	0.758	0.604	0.564	0.664	0.757	0.696	0.332	0.756	0.757	0.757	0.343
20_intra							0.327	0.511	0.559	0.999	0.465	0.494	0.795	0.998	0.817	0.488	0.989	0.997	0.989	0.486
20_flux								0.764	0.551	0.328	0.565	0.485	0.384	0.327	0.408	0.191	0.329	0.328	0.330	0.169
20_connector									0.574	0.511	0.544	0.503	0.496	0.512	0.516	0.290	0.515	0.513	0.516	0.317
44_dIIC										0.560	0.960	0.779	0.642	0.559	0.681	0.259	0.576	0.560	0.577	0.200
44_intra											0.465	0.495	0.795	0.998	0.818	0.488	0.989	0.997	0.989	0.487
44_flux												0.712	0.598	0.465	0.638	0.236	0.484	0.466	0.485	0.158
44_connector													0.528	0.495	0.550	0.382	0.496	0.495	0.497	0.274
100_dIIC														0.795	0.964	0.590	0.815	0.794	0.816	0.399
100_intra															0.817	0.488	0.988	0.995	0.988	0.487
100_flux																0.478	0.839	0.817	0.841	0.378
100_connector																	0.485	0.487	0.485	0.484
1000_dIIC																		0.987	0.998	0.488
1000_intra																			0.998	0.486
