# Supplementary material for: Connectivity assessment and prioritization of urban grasslands as a helpful tool for effective management of urban ecosystem services
Source: PLoS One. 2020 Dec 28;15(12):e0244452. doi: 10.1371/journal.pone.0244452 (PMC7769447; doi:10.1371/journal.pone.0244452)
Supplement: S1 File — The green color shows the 20% of grasslands patches with the highest dIIC values for a particular dispersal distance; the gray indicates the remaining 80% of patches. The maps are ordered according to increased assumed dispersal distances thresholds: 2m, 20 m, 44 m, 100 m and 1000 m. (PDF) [file pone.0244452.s002.pdf]

S1 File. The high-resolution maps of location of urban grassland patches in Wrocław city. The green color shows the 20% of grasslands patches with the highest dIIC values for a particular dispersal distance; the gray indicates the remaining 80% of patches. The maps are ordered according to increased assumed dispersal distances thresholds: 2m, 20 m, 44 m, 100 m and 1000 m.

S1A Map. Location of urban grassland patches in Wrocław city. The green color shows the 20% of grasslands patches with the highest dIIC values for a dispersal distance of 2 m; the gray indicates the remaining 80% of patches. The shapefiles are provided by [66,69].

S1B Map. Location of urban grassland patches in Wrocław city. The green color shows the 20% of grasslands patches with the highest dIIC values for a dispersal distance of 20 m; the gray indicates the remaining 80% of patches. The shapefiles are provided by [66,69].

S1C Map. Location of urban grassland patches in Wrocław city. The green color shows the 20% of grasslands patches with the highest dIIC values for a dispersal distance of 44 m; the gray indicates the remaining 80% of patches. The shapefiles are provided by [66,69].

S1D Map. Location of urban grassland patches in Wrocław city. The green color shows the 20% of grasslands patches with the highest dIIC values for a dispersal distance of 100 m; the gray indicates the remaining 80% of patches. The shapefiles are provided by [66,69].

S1E Map. Location of urban grassland patches in Wrocław city. The green color shows the 20% of grasslands patches with the highest dIIC values for a dispersal distance of 1000 m; the gray indicates the remaining 80% of patches. The shapefiles are provided by [66,69].

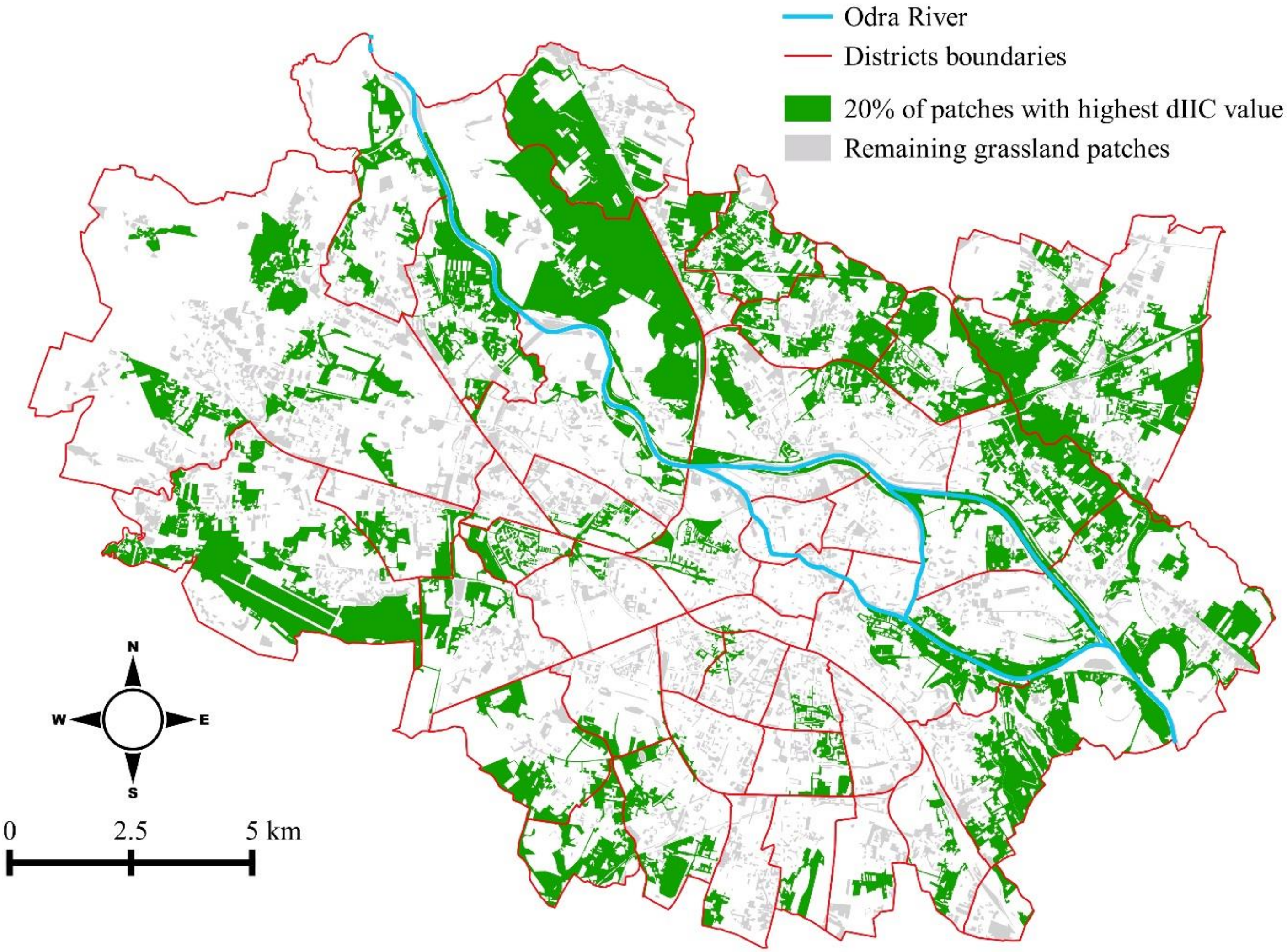

S1A Map. Location of urban grassland patches in Wrocław city. The green color shows the 20% of grasslands patches with the highest dIIC values for a dispersal distance of **2 m**; the gray indicates the remaining 80% of patches. The shapefiles are provided by [66,69].

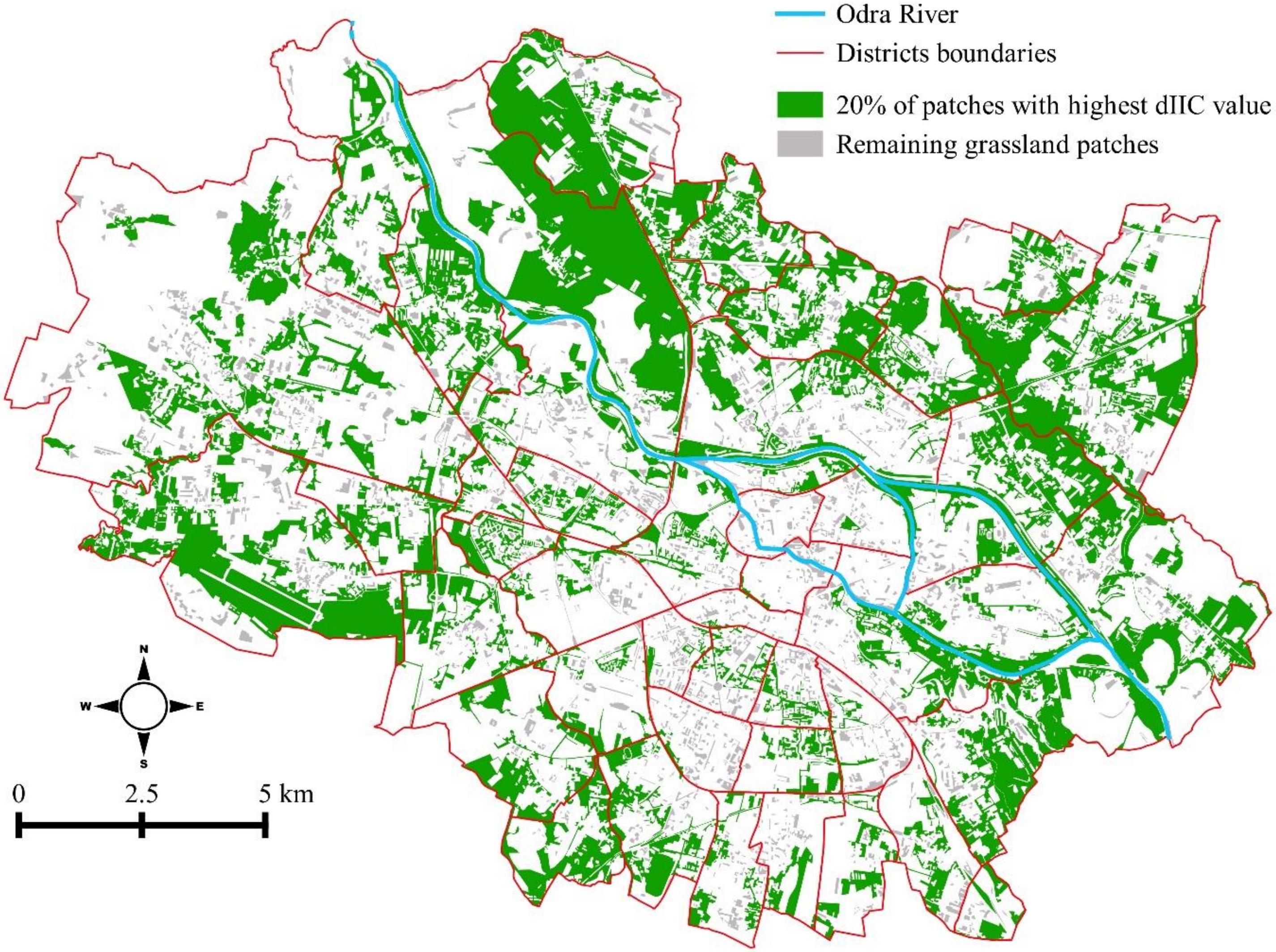

S1B Map. Location of urban grassland patches in Wrocław city. The green color shows the 20% of grasslands patches with the highest dIIC values for a dispersal distance of **20 m**; the gray indicates the remaining 80% of patches. The shapefiles are provided by [66,69].

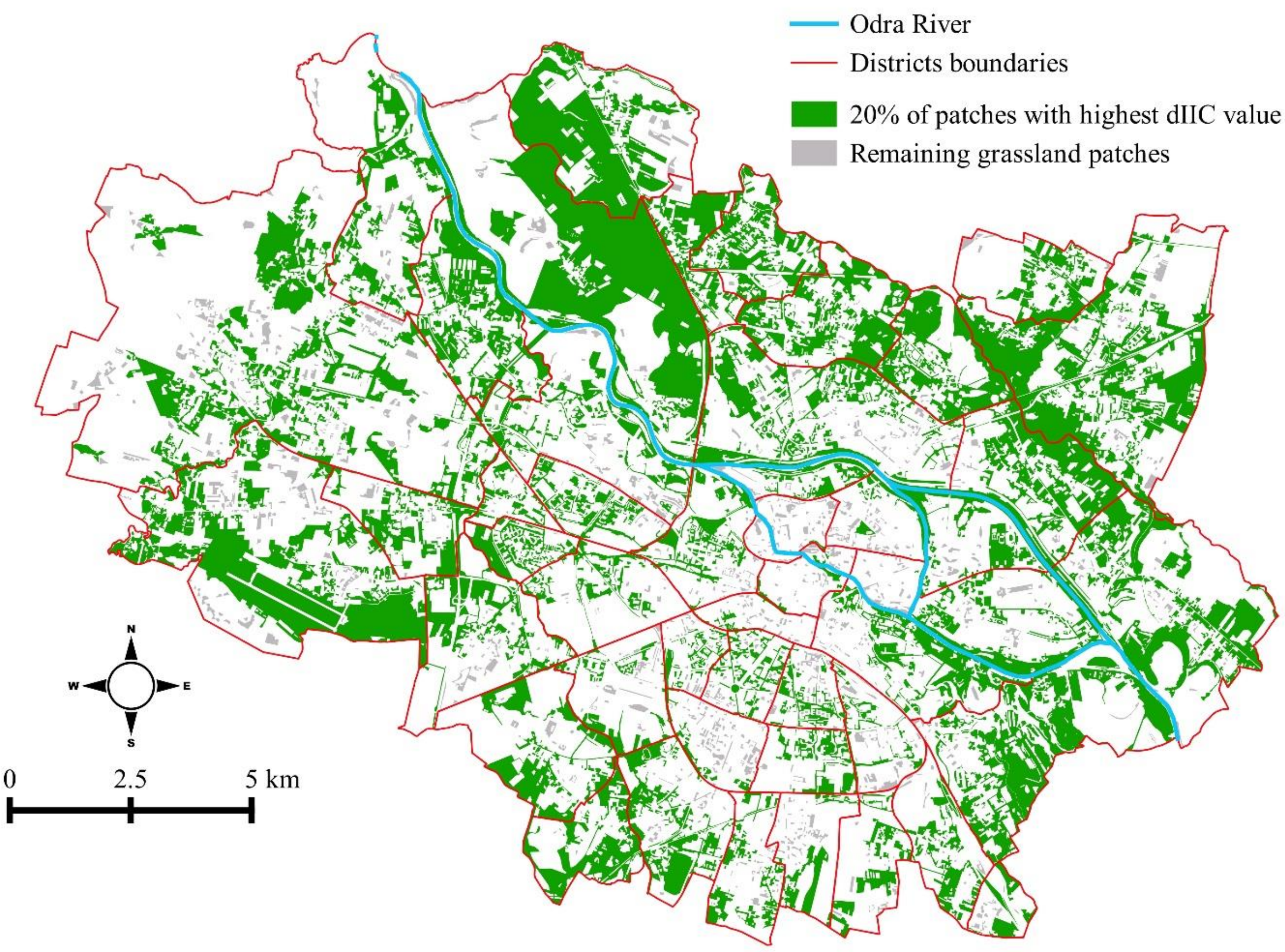

S1C Map. Location of urban grassland patches in Wrocław city. The green color shows the 20% of grasslands patches with the highest dIIC values for a dispersal distance of **44 m**; the gray indicates the remaining 80% of patches. The shapefiles are provided by [66,69].

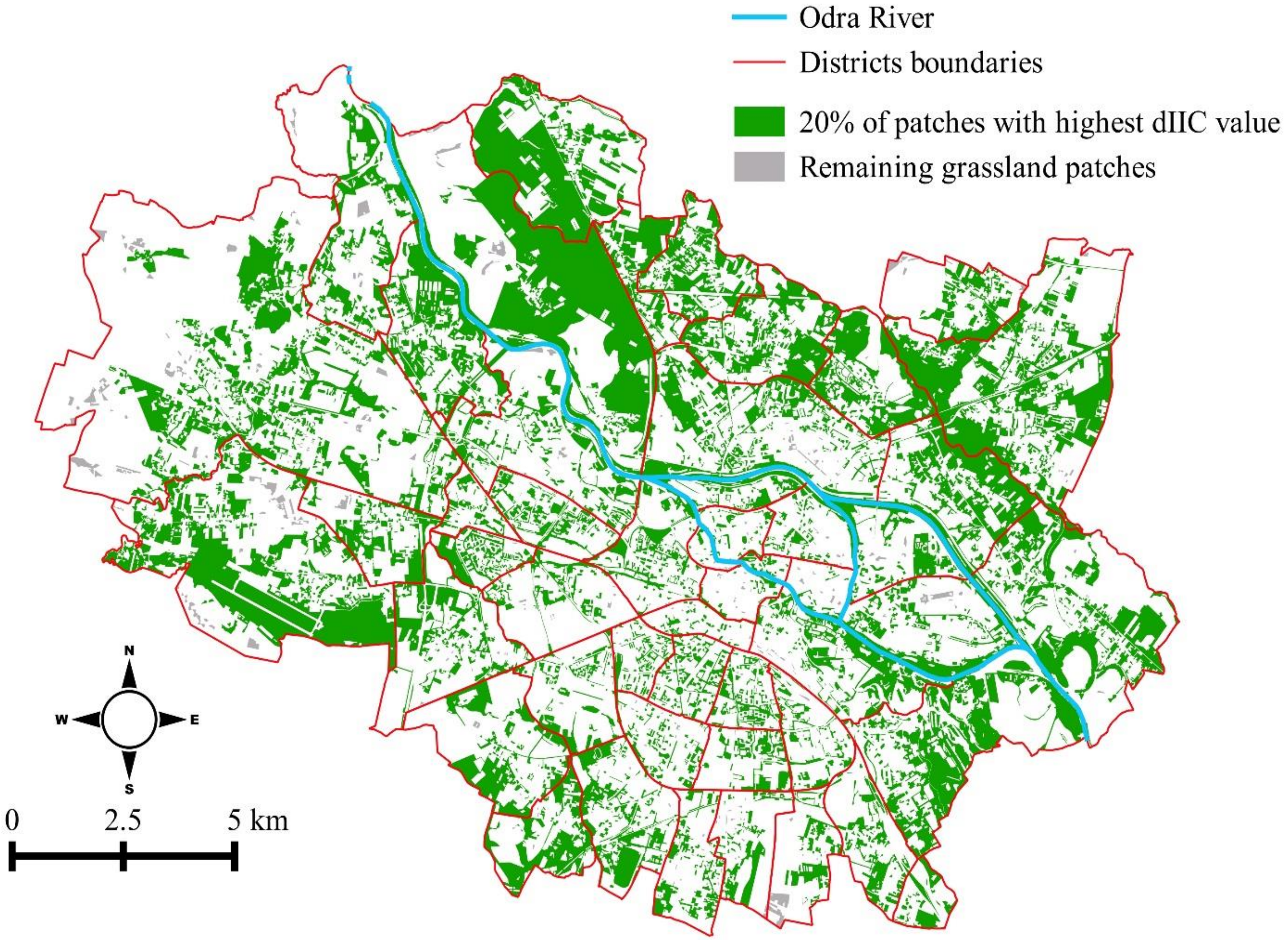

S1D Map. Location of urban grassland patches in Wrocław city. The green color shows the 20% of grasslands patches with the highest dIIC values for a dispersal distance of **100 m**; the gray indicates the remaining 80% of patches. The shapefiles are provided by [66,69].

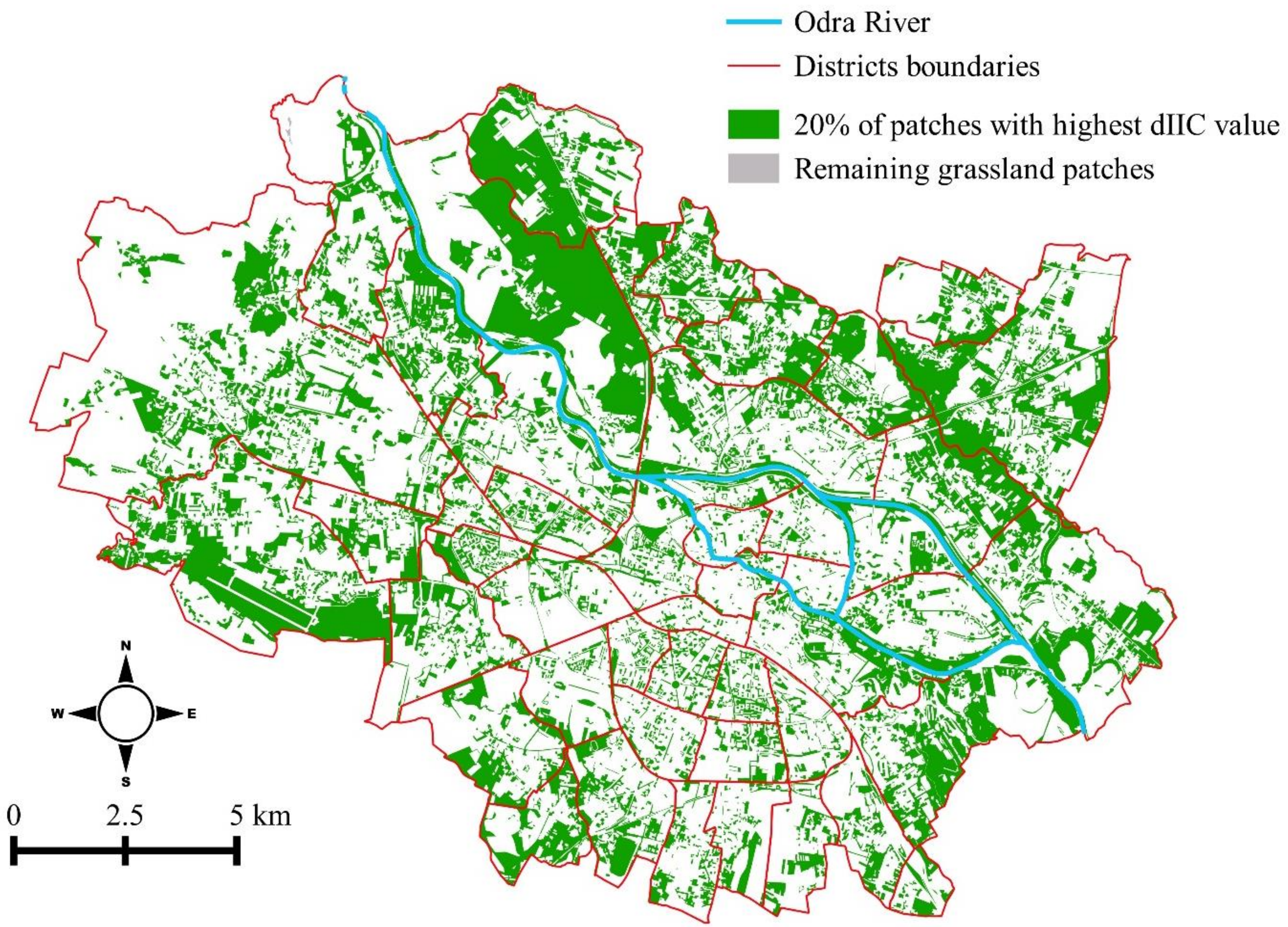

S1E Map. Location of urban grassland patches in Wrocław city. The green color shows the 20% of grasslands patches with the highest dIIC values for a dispersal distance of **1000 m**; the gray indicates the remaining 80% of patches. The shapefiles are provided by [66,69].
