# Supplementary material for: Connectivity assessment and prioritization of urban grasslands as a helpful tool for effective management of urban ecosystem services
Source: PLoS One. 2020 Dec 28;15(12):e0244452. doi: 10.1371/journal.pone.0244452 (PMC7769447; doi:10.1371/journal.pone.0244452)
Supplement: S2 File — The shapefiles are provided by [66, 69]. (PDF) [file pone.0244452.s003.pdf]

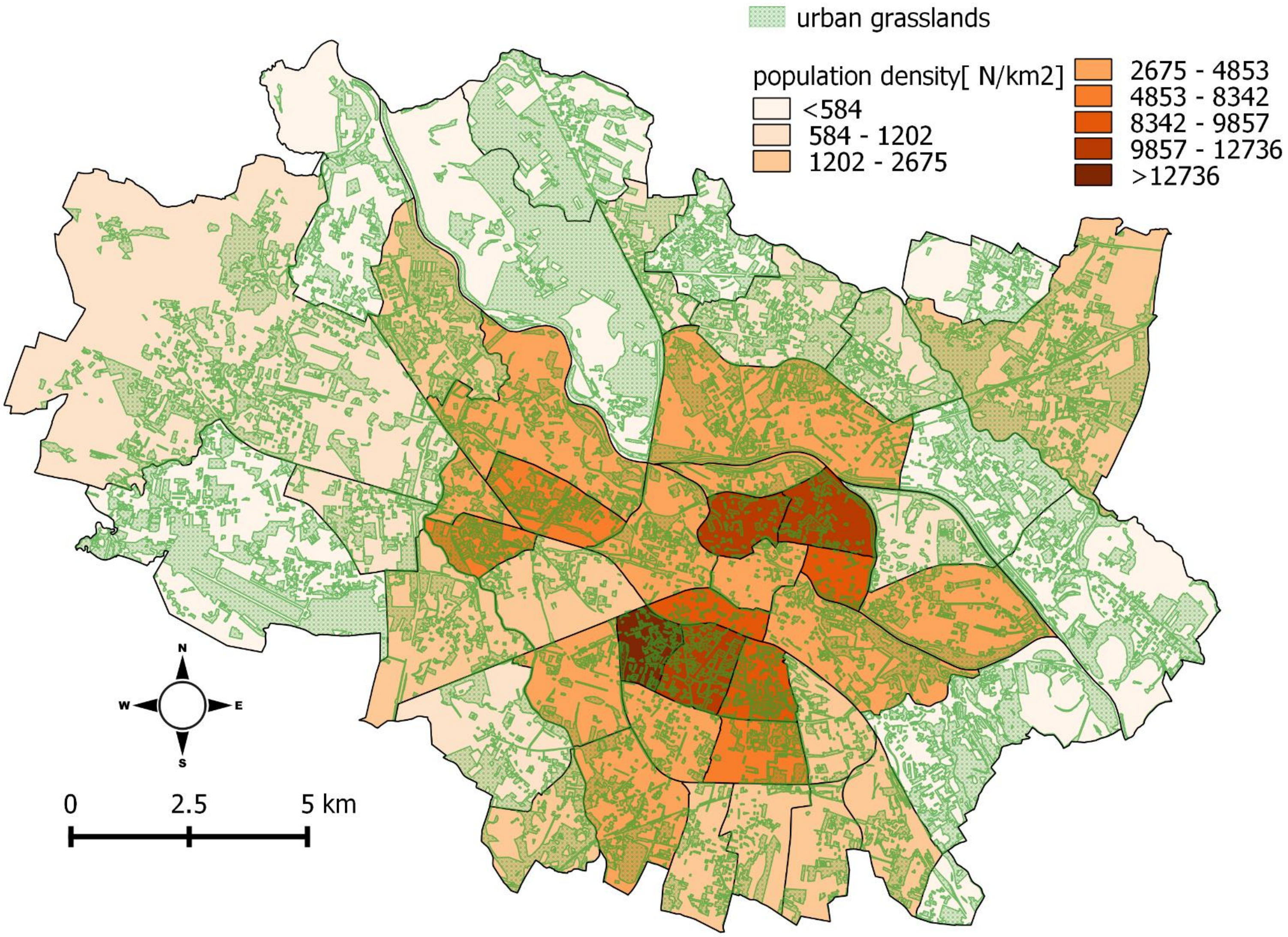

S2 File. Distribution of urban grassland patches against a background of human population density in districts of Wrocław city. The shapefiles are provided by [66,69].
